# Multiple binding modes of a small molecule to human Keap1 revealed by X-ray crystallography and molecular dynamics simulation

**DOI:** 10.1016/j.fob.2015.06.011

**Published:** 2015-06-30

**Authors:** Mikiya Satoh, Hajime Saburi, Tomoyuki Tanaka, Yoshinori Matsuura, Hisashi Naitow, Rieko Shimozono, Naoyoshi Yamamoto, Hideki Inoue, Noriko Nakamura, Yoshitaka Yoshizawa, Takumi Aoki, Ryuji Tanimura, Naoki Kunishima

**Affiliations:** a Bio-Specimen Platform Group, RIKEN SPring-8 Center, 1-1-1 Kouto, Sayo-cho, Sayo-gun, Hyogo 679-5148, Japan; b Pharmaceutical Research Laboratories, Toray Industries, Inc., 10-1, Tebiro 6-chome, Kamakura, Kanagawa 248-8555, Japan

**Keywords:** DTT, dithiothreitol, Keap1, Kelch-like ECH-associated protein 1, MD, molecular dynamics, Nrf2, Nuclear factor erythroid 2-related factor 2, PDB, Protein Data Bank, Oxidative stress, Antioxidant response, β-Propeller, Crystal packing, Fragment-based drug discovery, Structure-based drug design

## Abstract

Keap1 protein acts as a cellular sensor for oxidative stresses and regulates the transcription level of antioxidant genes through the ubiquitination of a corresponding transcription factor, Nrf2. A small molecule capable of binding to the Nrf2 interaction site of Keap1 could be a useful medicine. Here, we report two crystal structures, referred to as the soaking and the cocrystallization forms, of the Kelch domain of Keap1 with a small molecule, Ligand1. In these two forms, the Ligand1 molecule occupied the binding site of Keap1 so as to mimic the ETGE motif of Nrf2, although the mode of binding differed in the two forms. Because the Ligand1 molecule mediated the crystal packing in both the forms, the influence of crystal packing on the ligand binding was examined using a molecular dynamics (MD) simulation in aqueous conditions. In the MD structures from the soaking form, the ligand remained bound to Keap1 for over 20 ns, whereas the ligand tended to dissociate in the cocrystallization form. The MD structures could be classified into a few clusters that were related to but distinct from the crystal structures, indicating that the binding modes observed in crystals might be atypical of those in solution. However, the dominant ligand recognition residues in the crystal structures were commonly used in the MD structures to anchor the ligand. Therefore, the present structural information together with the MD simulation will be a useful basis for pharmaceutical drug development.

## Highlights

• Keap1 is useful target for the design of drugs that regulate the response to oxidative stresses.
• We determined two complex crystal structures of Keap1 with a small molecule ligand.
• The ligand binds to Keap1 so as to mimic the physiological substrate Nrf2.
• From molecular dynamics simulation results, the binding modes observed may be atypical in solution.
• Key residues for ligand binding are common between crystal and MD structures.

## Graphical abstract

**Figure:**
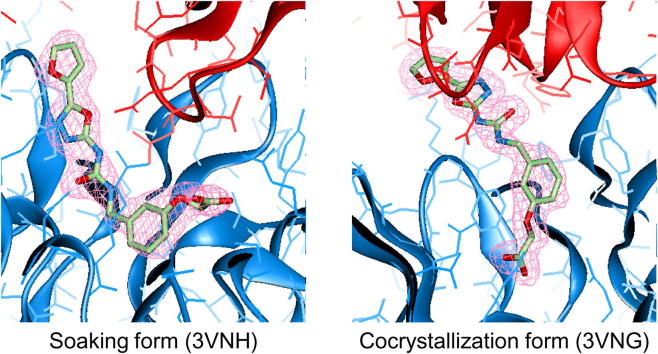

## 1 Introduction

Living organisms have specific defense systems against various environmental stresses. Of all these stresses, the oxidative stress have been a focus of constant attention in medicine because it is related to many pathologies including cancer [1,2], cardiovascular disease [3,4], diabetes [5,6], neurodegenerative disease [7,8], chronic arthritis [9,10] and aging [11,12]. Understanding of the antioxidant response system is important to develop medical treatments for these pathologies [13]. The antioxidant response is accomplished by the sensing of oxidants and the subsequent activation of antioxidant genes [14]. The sensing of oxidants such as reactive oxygen species and electrophilic xenobiotics is carried out by the Kelch-like ECH-associated protein 1 (Keap1) [15]. On the other hand, the nuclear factor erythroid 2-related factor 2 (Nrf2) protein [16] is responsible for the transcriptional regulation of about 200 antioxidant proteins including many enzymes/transporters for drug metabolism and Nrf2 itself [17]. Keap1 is susceptible to a posttranslational modification by oxidants at certain reactive cysteine residues, allowing Keap1 to sense them [18,19]. Keap1 and other proteins assemble to make the Cullin 3-based E3 ubiquitin ligase complex that binds and ubiquitinates Nrf2. After the ubiquitination, Nrf2 is rapidly degraded by proteasome to keep the lower level of intracellular Nrf2, and therefore the transcription of antioxidant genes is suppressed in the quiescent cells [19,20]. The oxidative-stress-induced modification of Keap1 at specific cysteine residues inhibits the Nrf2 ubiquitination, thereby elevating the intracellular Nrf2 level [19]. As a result, the intact Nrf2 activates the transcription of antioxidant genes through making a complex with the small Maf protein and subsequent binding to a cis-acting DNA sequence termed the antioxidant response element located in the promoter regions of target genes [16].

The Keap1 molecule mainly consists of the N-terminal domain, the C-terminal Kelch domain and the intervening region located in-between the two domains. A single particle analysis of electron microscopy confirmed that Keap1 in solution state forms a homodimer assembled at two cognate N-terminal domains [21]. The N-terminal domain also mediates interactions with other components of the Cullin 3-based E3 ubiquitin ligase complex [22], and contains a cysteine residue Cys151 that senses the oxidative stress [19]. The other sensing cysteine residues Cys273 and Cys288 present in the intervening region [18]. The Kelch domain is responsible for the interaction with Nrf2. As the binding site to Keap1, the ETGE motif located in the N-terminal region of Nrf2 was found first [23]. The DLG motif in the N-terminal region was identified later, as the second binding site required for the ubiquitination/degradation of Nrf2 [24]. Biophysical analyses using nuclear magnetic resonance and isothermal titration calorimetry revealed that the DLG and ETGE motifs interact independently with the Kelch domain by different dissociation constants of 0.5 μM and 8 nM, respectively [25]. Based on analyses in molecular biology, McMahon et al. proposed a two-site interaction model so-called “tethering” mechanism in which two Kelch domains of the Keap1 dimer recognize a single molecule of Nrf2 to facilitate the Cullin-mediated ubiquitination of Nrf2 [26].

These studies imply that a small molecule capable of binding to the Nrf2 interaction site on the Keap1 Kelch domain could be a useful medicine to activate the cellular defense to the oxidative stress through inhibiting the ubiquitination of Nrf2. Although several candidates for such compounds were reported, no one was applied in practical use [27–29]. Precise structural information, for instance, from the X-ray crystallography at high resolution, is indispensable for the structure-based drug design. To date, several crystal structures were reported: the Kelch domain [30], the Kelch domain in complex with the Nrf2 peptide containing the ETGE motif [31,32] or the DLG motif [33,34]. However, structural information of Keap1 complexes with small molecule ligands is still limited; only two complex structures have been reported recently [35,36]. Structural comparison between multiple crystal structures of Keap1 complexes with different small molecule ligands would be useful for the more effective design of Keap1 inhibitors [28]. Here we determined a crystal structure of the Kelch domain of human Keap1 in complex with a small ligand referred to as Ligand1 that has a novel chemical scaffold. Interestingly, two different binding modes were observed in the Keap1–Ligand1 complex crystals.

## 2 Results and discussion

### 2.1 Screening and characterization of Ligand1

**Fig. 1:**
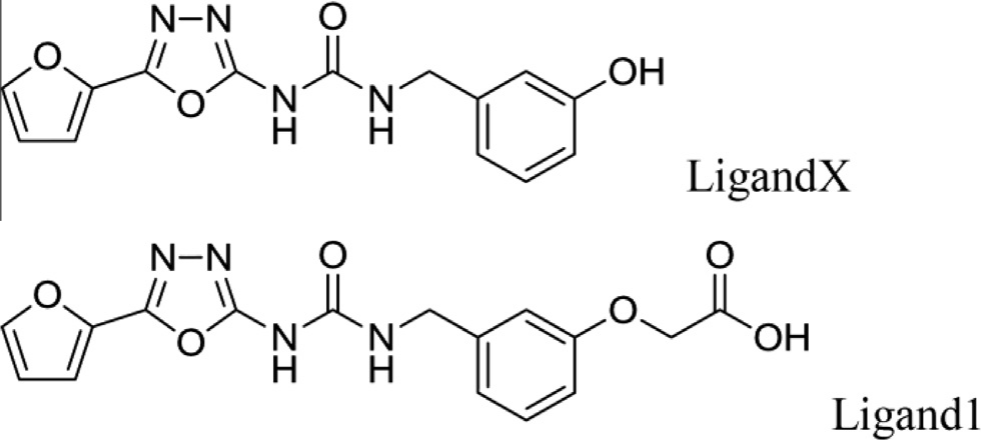
Chemical structure of ligands.

To search for the candidate compounds, an *in silico* screening was performed on the crystal structure of the Kelch domain of human Keap1 in complex with the ETGE peptide of Nrf2 (PDB entry 2flu) [32]. The in-house and commercially available compounds were docked against the Nrf2 peptide binding site. Based on the docking score and the predicted affinity against Keap1, 65 compounds were selected. Then, the binding ability of these compounds was evaluated using a surface plasmon resonance-based solution assay at 50 μM of the compound concentration. We found 27 active compounds including the LigandX (Fig. 1).

**Fig. 2:**
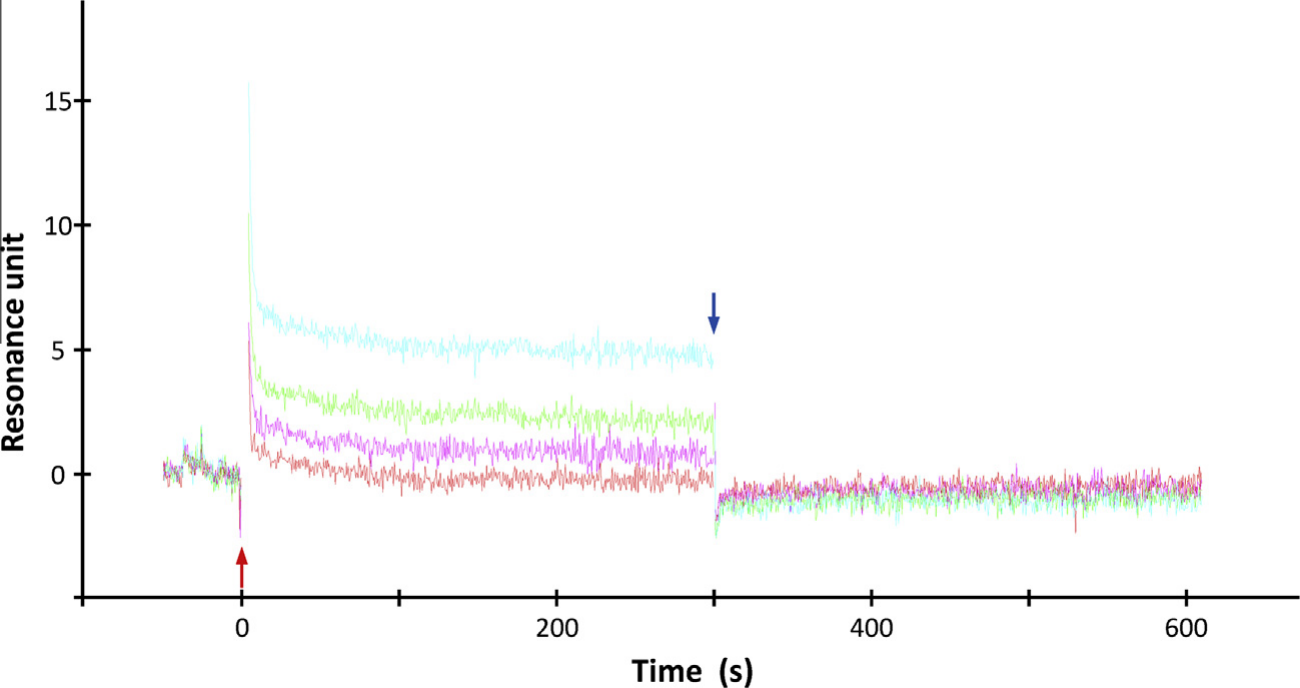
Surface plasmon resonance-based solution experiment to evaluate the Keap1–Ligand1 interaction. The sensorgram from the immobilized GST fusion of Keap1 Kelch domain on the sensor chip with four different concentrations of Ligand1 after the bulk solvent correction and reference correction using GST is shown. The color codes used were: red for 12.5 μM, magenta for 25 μM, green for 50 μM and sky blue for 100 μM. The starting and the stopping points of the ligand addition are indicated by red and blue arrows, respectively.

Based on the structural comparison between the docking pose of LigandX and the ETGE peptide of Nrf2 in 2flu, we designed and synthesized the Ligand1 in which the phenol moiety of LigandX was modified by the oxyacetic acid group that intended to mimic the sidechain of the first (N-terminal) glutamic acid residue in the ETGE peptide (see Section 4 for the synthesis of Ligand1). The association constant of Ligand1 to the human Keap1 Kelch domain was estimated as the third to the fourth power of ten from the equilibrium affinity analysis of a surface plasmon resonance-based solution experiment (Fig. 2). Unfortunately, the limited solubility of Ligand1 (less than 1 mM) hampered further analyses on binding properties including the precise association constant, the number of binding sites, and the cooperativity. The Keap1–Ligand1 interaction was also confirmed by another assay using AlphaScreen (PerkinElmer Inc.), a bead-based, amplified luminescent proximity homogeneous assay, which revealed the competitive effect of Ligand1 on the interaction between the Nrf2 peptide and the Kelch domain of Keap1 (Supplemental Fig. S1).

### 2.2 Quality of crystal structures

Two crystal structures of the human Keap1 Kelch domain in complex with Ligand1, the soaking form and the cocrystallization form, were determined at 2.1 Å resolution (Table 1). In these two forms, the asymmetric unit contained a Kelch domain and a Ligand1 molecule. The final models of the Kelch domain covered the amino-acid residues 322–609 with well-defined electron densities, while the N-terminal 21 residues (20 His-tag residues and Ala321) were not included due to a structural disorder, as observed in the crystal structure of the same Kelch domain reported (PDB entry 1u6d) [30]. The temperature factor (*B*) values calculated from final models (average) were comparable to the Wilson *B* values from corresponding diffraction data. The soaking form crystal was isomorphous to 1u6d with the space group *P*6_5_22, whereas the cocrystallization form revealed a different crystal packing with the other space group *P*2_1_2_1_2_1_. The stereochemistry analysis revealed no residue in generously allowed or disallowed regions of the Ramachandran plot, except for Arg336 and His516 in the generously allowed region. These two residues were found in well-defined electron densities without steric clashes. All atoms comprising Ligand1 were identified in electron density maps with reasonable individual *B* values comparable to those of neighboring protein atoms, indicating high occupancy of the ligand (Table 1). Thus we fixed the occupancies of all atoms comprising Ligand1 to 1.0 and did not refine them. In addition, annealed 2*F*_o_−*F*_c_ omit maps for Ligand1 in both the forms provided clear electron densities comparable to those of corresponding final 2*F*_o_−*F*_c_ maps (Supplemental Fig. S2), confirming the existence of ligands bound.

### 2.3 Recognition of Ligand1

The overall structural architecture of the human Keap1 Kelch domain in our structures is the same as that in the PDB entry 1u6d that represents the β-propeller structure composed of a sixfold repeat of all β domain called “blade” [30]. The Rmsd values from the superposition of C^α^ atoms (residues 325–609) between 1u6d and our structures are 0.241 Å for the soaking form and 0.431 Å for the cocrystallization form. The latter value is similar to that from the C^α^ superposition between the soaking and the cocrystallization forms: 0.449 Å. Reflecting the crystal packing difference, the N-terminal three residues adopt totally different main-chain conformations in the cocrystallization form when compared to other forms, thereby excluding these residues in the superposition. Elsewhere, the cocrystallization form is similar to the others in terms of the main-chain structure. On the other hand, 1u6d and the soaking form confer essentially the same main-chain structure including the N-terminal region.

**Fig. 3:**
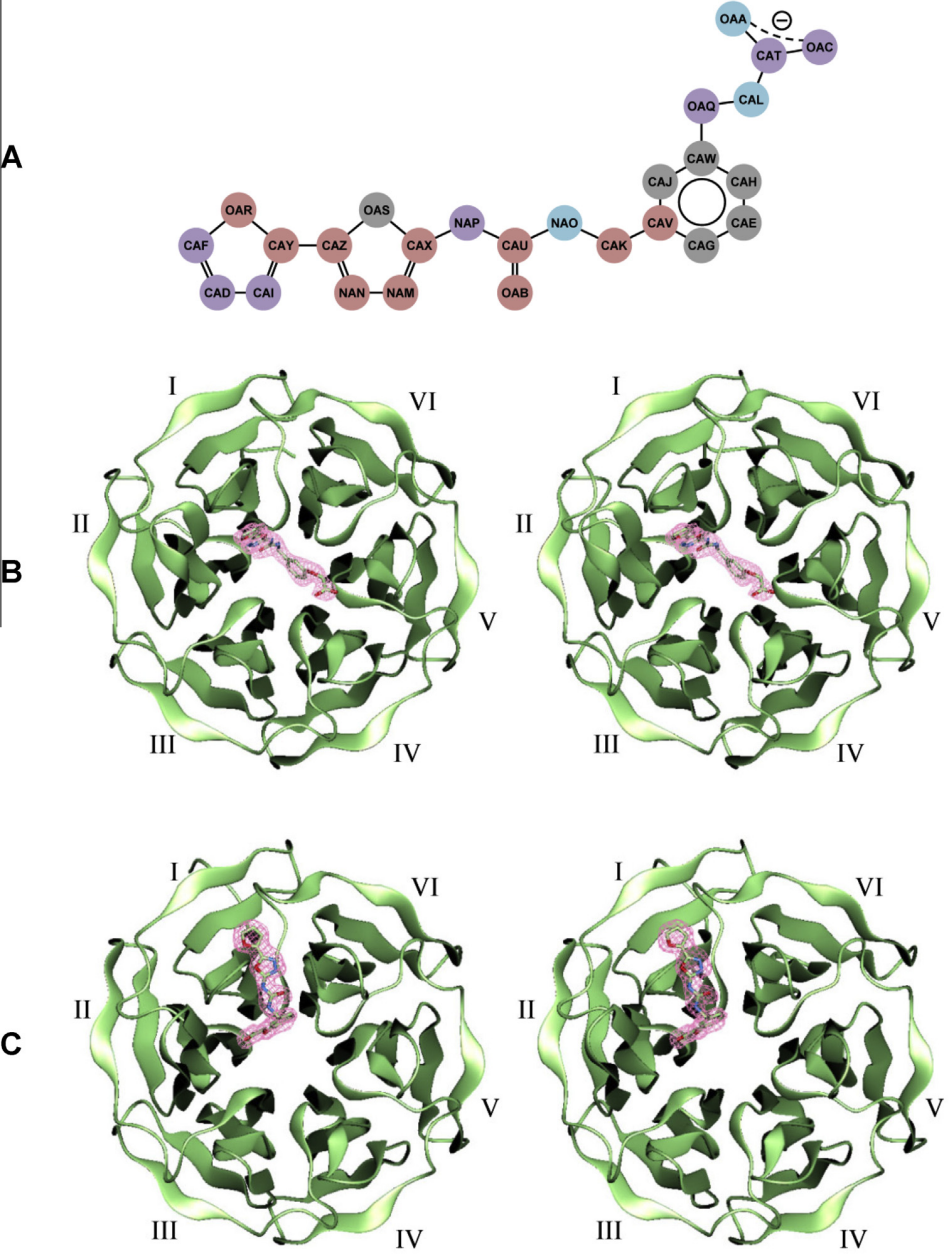
Structure of Keap1 in complex with Ligand1. (A) Schematic representation of Keap1–Ligand1 interactions. The notation of atoms is the same as that used in the PDB entries 3vng and 3vnh. The atom names beginning C, N and O denote carbon, nitrogen and oxygen atoms, respectively. The atoms recognized by Keap1 in the soaking form, in the cocrystallization form and in both the forms are colored red, blue and purple, respectively. Unrecognized atoms are colored gray. (B) Stereo representation of the soaking form structure. The Keap1 domain and the Ligand1 molecule are depicted as a ribbon drawing and a stick model, respectively. The blades of β-propeller are labeled. The final 2*F*_o_ − *F*_c_ electron densities at 2.1 Å resolution contoured at 1*σ* are shown around the Ligand1 model. (C) Cocrystallization form structure shown in the same manner as (B).

When the soaking and the cocrystallization forms are compared, the Ligand1 molecule is found on the same side of the 6-bladed β-propeller that is opposite to the N- and C-termini. However, the precise locations of two binding sites are distinct (Fig. 3B and C). In the soaking form, the Ligand1 molecule approximately locates on the central hole of Keap1. On the other hand, in the cocrystallization form, the binding site of Ligand1 relocates by about 10 Å toward the first blade. The Ligand1 molecule is recognized by ten residues in the soaking form and seven residues in the cocrystallization form (Table 2). These residues are well-conserved in the Keap1 members from various organisms. Interestingly, some of these residues are overlapped, indicating an apparent multiple recognition mode to the same ligand (Fig. 3A). The residues used in both the forms are Tyr334, Ser363, Arg380, Asn382 and Asn414. On the other hand, the form-specific recognition residues are Arg415, Arg483, Ser508, Ala556 and Gly603 for the soaking form, whereas Arg336 and Ser602 for the cocrystallization form. Interaction types used are: 1 hydrogen bond, 1 water-mediated hydrogen bond, 3 electrostatic interactions and 19 nonpolar interactions for the soaking form; 5 hydrogen bonds, 3 water-mediated hydrogen bonds and 11 nonpolar interactions for the cocrystallization form. Thus, in terms of the interaction type, nonpolar interactions and hydrogen bonds dominate in the soaking and the cocrystallization forms, respectively.

From a detailed comparison between 1u6d and the soaking form, a ligand-induced rearrangement of side-chain atoms is observed at several ligand recognition residues: Arg380, Asn382, Arg415, Arg483 and Ser508. On the other hand, from that between 1u6d and the cocrystallization form, another ligand-induced rearrangement is observed at a few ligand recognition residues: the side-chains of Arg336 and Arg380, and the main-/side-chain of Asn382. In addition, probably reflecting the crystal packing, several peripheral loops adopt slightly different main-/side-chain conformations. Of these peripheral loops, the β-hairpin protrusion of the second blade (residues 380–389) in which two ligand recognition residues Arg380 and Asn382 exist, shows a limited conformational change in the main-/side-chain structure, probably reflecting both the ligand binding and the crystal packing difference. Therefore, this β-hairpin in the second blade may be important for the ligand recognition. Among six β-hairpin protrusions of Keap1, only that of the first blade (residues 334–338) adopts relatively rare class 1 conformation [37] where two ligand recognition residues Tyr334 and Arg336 exist; other five β-hairpins adopt the class 2 conformation. Interestingly, the six β-hairpins of Keap1 are accompanied with similar uncommon β-bulges including the invariant glycine doublet, which cannot be classified by the current criteria [38] of β-bulge. In the soaking form, a ligand recognition residue Arg483 locates on the β-hairpin in the fourth blade (residues 478–483). Since they harbor the ligand binding residues, the β-hairpins in the first and the fourth blades may also be important for the ligand recognition, although they do not show large conformational differences in the main-chain structure upon binding ligand.

### 2.4 Structural comparison between Ligand1 and Nrf2

**Fig. 4:**
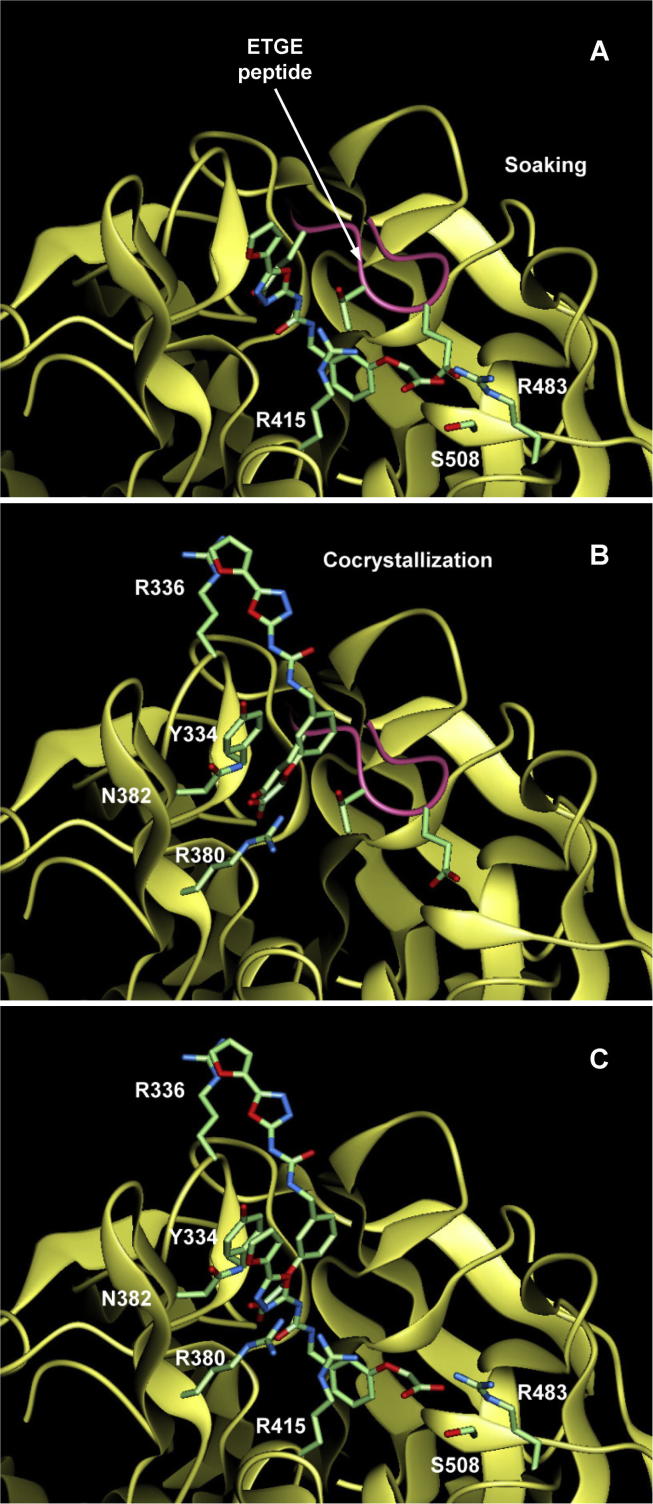
Structural comparisons. The Keap1 domain and the Ligand1 molecule are depicted as a ribbon drawing and a stick model, respectively. Representative residues for the ligand recognition are shown as stick models and labeled. (A) Ligand1 in the soaking form compared with the ETGE peptide. The ETGE peptide from the PDB entry 1x2r[31] is superposed and shown in a magenta wire model with side-chain stick models for the first glutamate at bottom, the threonine at middle and the second glutamate at top. (B) Ligand1 in the cocrystallization form compared with the ETGE peptide. The ETGE peptide superposed is shown in the same manner as (A). (C) Superposition between the soaking and the cocrystallization forms.

To understand the binding ability of Ligand1 to Keap1, present two Keap1–Ligand1 complexes were compared with other reported Keap1 complex structures that contain peptides from the physiological ligand, Nrf2. The clearest result was obtained from the Keap1 complex structure with the ETGE peptide of Nrf2: 1x2r [31], 2flu [32] and 3zgc [39]. When the soaking and the cocrystallization forms are superimposed onto 1x2r at corresponding C^α^ atoms, the carboxylate group of Ligand1 overlap with the first and the second glutamate of the ETGE motif, respectively (Fig. 4A and B). The other ETGE complexes, 2flu and 3zgc, provided essentially the same results. In another reported Keap1 complex structure 3wn7 [34] containing the DLG peptide of Nrf2, the binding mode of the DLG peptide was dissimilar to that of Ligand1. Accordingly, the superposition of the soaking and the cocrystallization forms clearly mimics the recognition mode of the ETGE peptide by Keap1 (Fig. 4C). This result is consistent with the solution experiment using AlphaScreen (PerkinElmer Inc.) in which Ligand1 inhibits the Keap1–ETGE binding (Supplemental Fig. S1). Other peptides from p62 [40] or prothymosin α [41] with essentially the same binding mode as that of Keap1–ETGE may compete with Ligand1 too. In addition, the overlapping of binding sites for the ETGE and DLG peptides indicates that Ligand1 can inhibit the Keap1–DLG binding partly.

This observation is similar to that in the first report on the Keap1 complex with small molecules by Marcotte et al. (PDB entries 4iqk and 4in4) [35] in which the sulfone group of small molecules located near to the acidic sidechains of the ETGE peptide when the Keap1–peptide complex was superimposed. However, the degree of mimicry seems to be higher in our structures where the recognition mode for the carboxylate group of Ligand1 by the Keap1 residues is corresponding exactly to that for the glutamate sidechains of the ETGE peptide. In the other reported Keap1 complex with small molecules by Jnoff et al. (PDB entries 4n1b, 4l7c and 4l7d) [36], the binding mode of the small molecule is dissimilar to that in our structures.

### 2.5 Molecular dynamics simulation

**Fig. 5:**
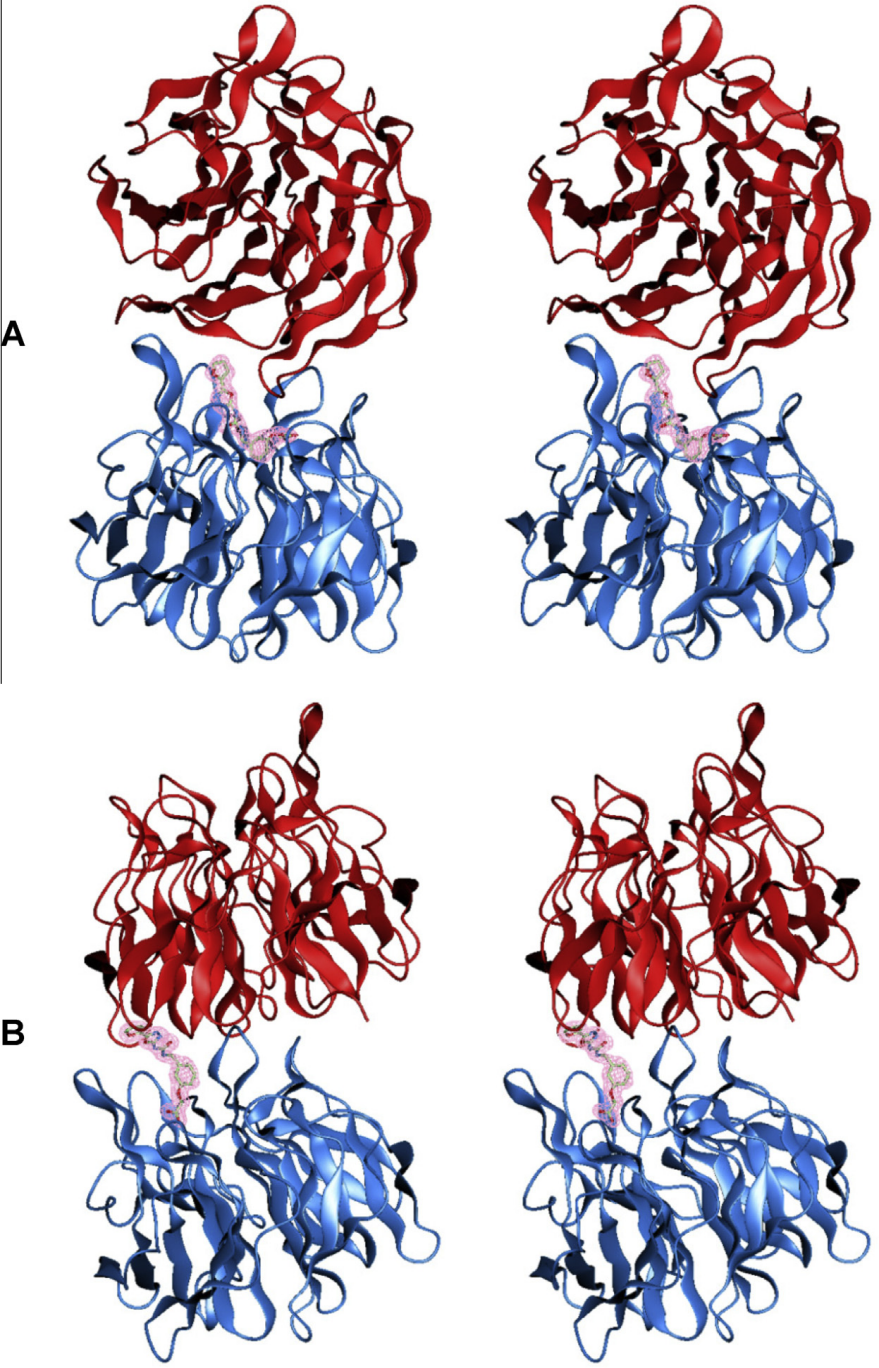
Stereo representations of crystal packing relevant to the ligand binding in the soaking form (A) and in the cocrystallization form (B). The Keap1 domain and the Ligand1 molecule are depicted as a ribbon drawing and a stick model, respectively. The asymmetric chain and the symmetry-related chain are colored blue and red, respectively. The final 2*F*_o_ − *F*_c_ electron densities at 2.1 Å resolution contoured at 1*σ* are shown around the Ligand1 model.

In the present structures, the Ligand1 bound to Keap1 mediates the crystal packing (Fig. 5). The numbers of contacts with interatomic distances not greater than 3.4 Å from the Ligand1 molecule to the asymmetric and the symmetry-related molecules of Keap1 are 20 and 5 for the soaking form, whereas 16 and 7 for the cocrystallization form, respectively. This indicates a substantial contribution of the crystal packing to the protein–ligand interactions in crystals. The crystal packing is known as a possible factor to influence on the structure-based drug design. For instance, the crystal contact at the active site can hamper binding ligands when the soaking method is used to prepare the complex crystals [42]. One of solutions to that situation is the crystal-contact engineering to obtain a new crystal form suitable for the ligand soaking experiments where the inappropriate crystal packing is disrupted by introducing mutations at the crystal contact [39]. However, the influence of crystal packing on the ligand binding mode when the packing is mediated by the ligand, is not fully investigated to date. Thus, to examine the influence of the crystal packing, we employed a molecular dynamics (MD) simulation in aqueous condition. For instance, such MD simulation was performed to rule out a false ligand which ejected from the binding pocket of the target protein within 50–100 ps of simulation [43]. In another example, an MD simulation for 20 ns was carried out to analyze the stability of a protein–ligand complex [44].

**Fig. 6:**
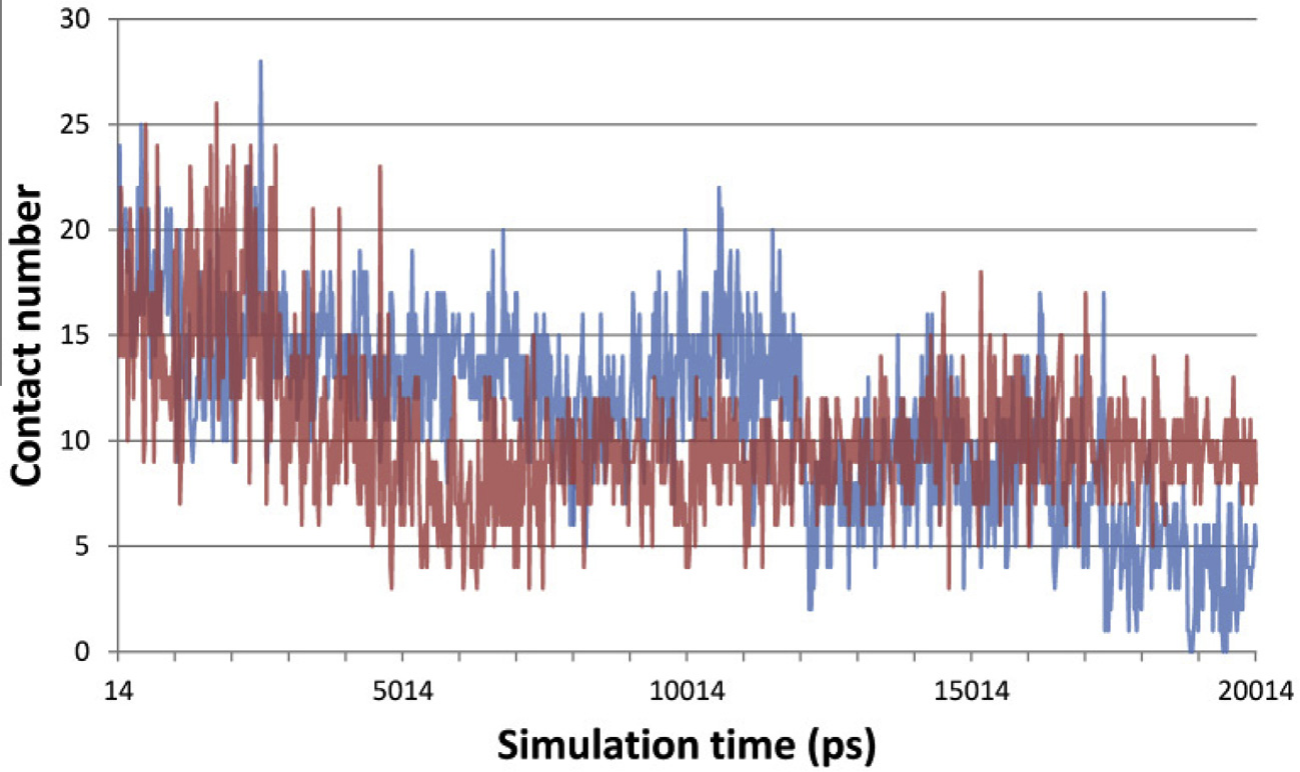
Protein–ligand contact number in MD trajectories. In a 20.014 ns MD trajectory, the protein–ligand contacts with interatomic distances not greater than 3.4 Å were counted and plotted versus time. Color codes used were red for the soaking form and blue for the cocrystallization form.

**Fig. 7:**
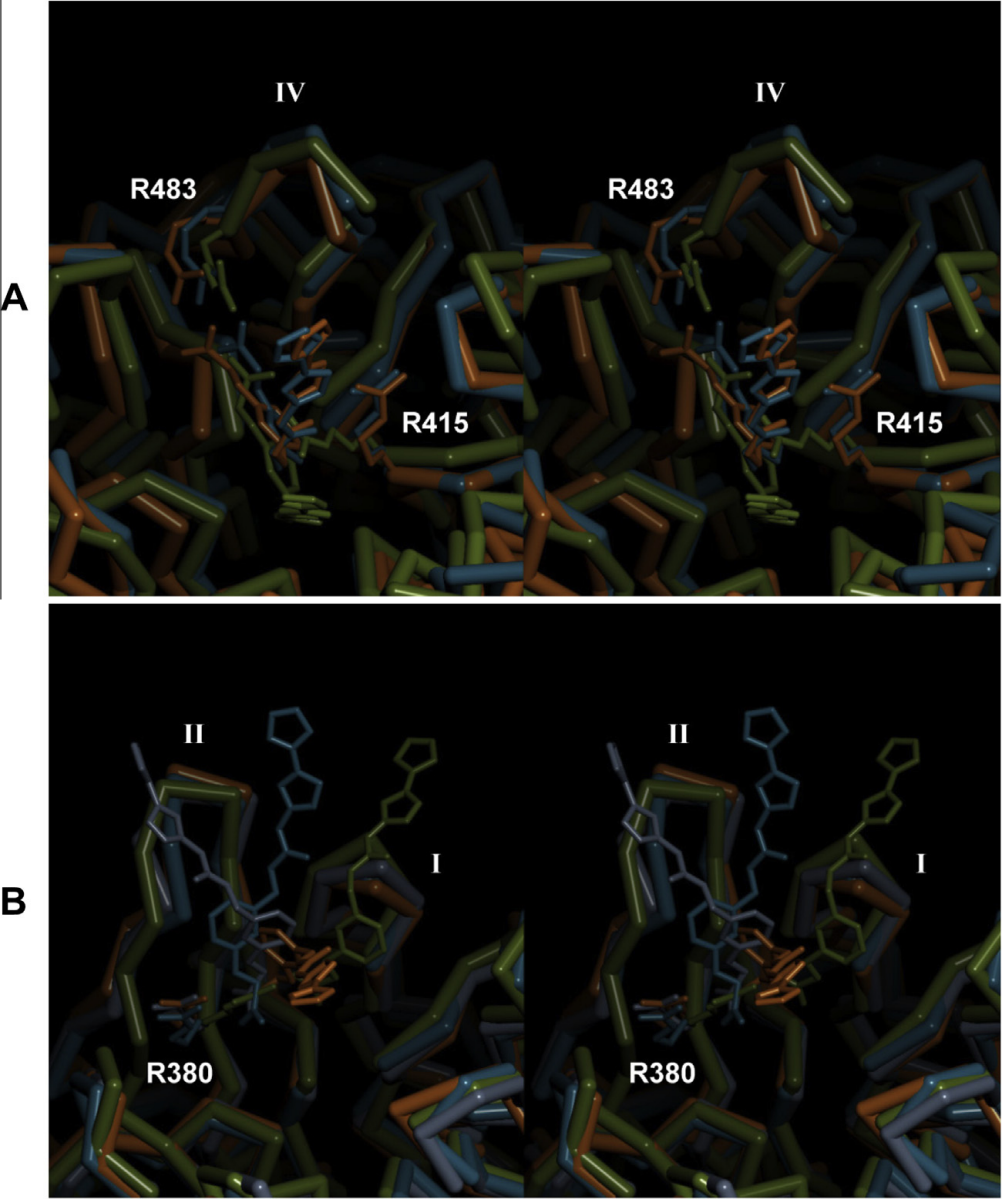
Stereo representations of MD structures from the soaking form (A) and from the cocrystallization form (B). The Keap1 domain and the Ligand1 molecule are depicted as a C^α^ trace and a stick model, respectively. Important residues for the ligand recognition are shown as stick models and labeled. The β-hairpins in the first, the second and the fourth blades are indicated as Roman numerals. In the 20.014 ns MD trajectories, only the center structure of each cluster is shown: the 892nd at 17.834 ns from the major cluster (blue) and the 774th at 15.474 ns from the minor cluster (orange) for the soaking form; the 658th at 13.154 ns from the major cluster (blue), the 842nd at 16.834 ns from the second cluster (orange) and the 597th at 11.934 ns from the third cluster (gray) for the cocrystallization form. These models are superimposed on the crystal structures (green) at corresponding backbone atoms.

For that reason, we performed a 20 ns MD simulation of present Keap1–Ligand1 complexes. The time course of protein–ligand contacts with interatomic distances not greater than 3.4 Å revealed that the contact number tends to decrease (Fig. 6). In the MD structures from the soaking form, the contact numbers achieved equilibria with the average number of about ten. However, in those from the cocrystallization form, several times of no contacts, indicating the dissociation of ligand, were observed in the later moments of the MD simulation. Judging from the time course of the Rmsd from the superposition of backbone atoms between the MD structures and the original crystal structure, the MD simulation revealed a metastable state after 10 ns (Supplemental Fig. S3). Thus, to classify the binding mode, 500 structures from 10 to 20 ns were submitted to a cluster analysis based on the method described by Daura et al. [45]. In this analysis, the structure with the highest number of neighbors is defined as the center of a cluster. As a result, MD structures in a trajectory could be classified into a few binding modes that were related to but distinct from those observed in the crystal structures (Fig. 7 and Table 3).

In the MD structures from the soaking form, two clusters sharing over 10% of 500 structures were obtained: the major cluster sharing 204 structures with the 892nd as the center and the minor cluster sharing 70 structures with the 774th as the center. The crystal structure does not belong to the major or minor clusters. In both the clusters, the guanidium groups of Arg415 and Arg483 interact with the ureido oxygen OAB and the carboxyl oxygens OAA/OAC of Ligand1, respectively. Thus, in the MD structures from the soaking form, Arg415 and Arg483 dominate the protein–ligand interactions. Difference between the two clusters is that Phe478 is used for the ligand recognition in the major cluster whereas Gly509 is used in the minor cluster (Table 3). On the other hand, in the MD structures from the cocrystallization form, three clusters sharing over 10% of 500 structures were obtained: the major cluster sharing 180 structures with the 658th as the center, the second cluster sharing 79 structures with the 842nd as the center, the third cluster sharing 63 structures with the 597th as the center. Again, the crystal structure does not belong to any of these clusters. In the major, the second, and the third clusters, the Ligand1 molecule locates in-between the β-hairpins of the first and the second blades, near the β-hairpin of the first blade, and near the β-hairpin of the second blade, respectively (Fig. 7B and Supplemental Fig. S4). The second cluster showed the lowest average contact number 8.34 per structure, indicating the weakest protein–ligand interactions in these three clusters (Table 3). A common feature of these clusters is that the guanidium group of Arg380 interacts with the carboxyl oxygens OAA/OAC of Ligand1, although Arg336 dominates the ligand recognition in the second cluster. Notably, the structural diversity of Ligand1 is higher in the cocrystallization form when compared to the soaking form.

From the results of the MD simulation, the binding modes observed in crystals seem to be atypical in the solution state, indicating that the MD simulation is required for the structure-based drug design when the ligand of interest mediates the crystal packing. However, importantly, a few residues for the ligand recognition, namely, Arg380, Arg415 and Arg483, are used commonly both in the crystal and the solution states. The binding modes observed in the MD structures from the soaking form have certain possibility to account for the moderate association constant of Ligand1 in solution, because the ligand binding was retained over 20 ns in the simulation. On the other hand, the binding modes observed in the MD structures from the cocrystallization form may be less stable. Presumably, in the cocrystallization form, the atypical binding mode appropriate for the crystal packing was selected in solution when the crystal nucleation occurred. This selection of minor and weak binding mode can occur in the case of other protein–ligand complexes with higher affinity.

## 3 Conclusions

To elucidate how Keap1 recognizes a small molecule ligand, we determined the complex crystal structure of Keap1–Ligand1 in two different forms. Because these two binding modes of Ligand1 mimic that of the physiological ligand Nrf2 peptide in different manners, the present structural information concomitant with the MD simulation will be a useful basis for the pharmaceutical drug development. For instance, the fragment-based drug discovery [46,47] based on the present results may contribute to design more potent inhibitors of Keap1, although the pharmacological efficacy of Ligand 1 needs to be examined elsewhere in future. At the same time, this work provides a lesson about the crystal packing effect that should be considered in the interpretation of protein–ligand complex structures. The MD simulation may be a good tool to investigate the crystal packing effect.

## 4 Materials and methods

### 4.1 Screening of compounds

The docking program *FRED* ver2.2 (OpenEye Scientific Software, Inc.) and the crystal structure of the Keap1 Kelch domain in complex with the Nrf2 peptide (PDB entry 2flu) were used in our *in silico* screening and the docking pose analysis of LigandX. The compounds in the *ZINC* database ver8 [48] and those in our in-house library were used for the *in silico* screening. The 3D coordinates of the library compounds were prepared by the program *LigPrep* (Schrödinger, LLC) and the 3D conformers for the docking were generated by the program *OMEGA* 2.3 (OpenEye Scientific Software, Inc.). The MASC consensus score in *FRED* was used for the compound selection.

### 4.2 Surface plasmon resonance-based equilibrium affinity analysis

To evaluate the Keap1–Ligand1 interaction, the surface plasmon resonance-based equilibrium affinity analysis was performed using Biacore S51 (Biacore AB/GE Healthcare). The protein sample used was the GST fusion of the human Keap1 Kelch domain. The Keap1 sample was immobilized on the Series S Sensor Chip CM5 (GE Healthcare) using the amine coupling kit (GE Healthcare). The experiment was performed at 298 K in a physiological solution [150 mM NaCl, 3.4 mM EDTA, 1% dimethylsulfoxide, 0.005% polysorbate 20, 10 mM HEPES buffer pH 7.4]. Data analysis was performed using the S51Evaluation program (GE Healthcare). The number of experiments was 2 for the zero concentration of Ligand1 and 1 for other concentrations. The resonance unit was calculated by subtracting the response on the sample sensor spot from that on the reference sensor spot with immobilized GST, and by considering a bulk correction to remove the difference in the refractive index of solvents [49]. The maximum value of resonance unit was estimated as 24 in the case of single binding site, based on the positive control experiment using the ETGE peptide of Nrf2. The affinity calculation using the steady state evaluation of *S51Evaluation* failed to calculate precise affinity but yielded a range of *K*_d_ value as more than 50 μM. A manual analysis using the double reciprocal plot based on the experimental data yielded a rough estimation of association constant as the third to the fourth power of ten depending upon the assumption on the binding site number.

### 4.3 Synthesis of Ligand1

#### 4.3.1 Synthesis of ethyl 2-(3-cyanophenoxy)acetate

To a solution of 3-hydroxybenzonitrile (1.429 g, 12 mmol) in acetone (120 ml), calcium carbonate (2.156 g, 15.8 mmol) and ethyl 2-bromoacetate (2.81 g, 18.8 mmol) were added. The mixture was stirred at room temperature for 11 h. The reaction mixture was filtered and the filtrate was evaporated under reduced pressure. The residue was purified by flash chromatography on silica gel to afford the product (2.46 g, 99%) as colorless oil.

^1^H-NMR (400 MHz, CDCL_3_) *δ*: 1.29 (3H, t, J = 7.8 Hz), 4.13 (2H, q, *J* = 7.8 Hz), 4.96 (2H, s), 7.27–7.48 (3H, m), 7.61 (1H, s).

ESI-MS: *m*/*z* = 206 (M+H)^+^.

#### 4.3.2 Synthesis of 2-(3-cyanophenoxy)acetic acid

To a solution of ethyl 2-(3-cyanophenoxy)acetate (616 mg, 3 mmol) in THF (12 ml), distilled water (6 ml) and 4 mM lithium hydroxide (3.75 ml) were added. The mixture was stirred at room temperature for 1 h. The mixture was diluted with 1 N hydrochloric acid to reach pH < 1 and extracted with dichloromethane (20 ml). The organic phase was washed with brine (20 ml), dried over with magnesium sulfate, and evaporated *in vacuo* to give the desired product (531 mg, 99%) as white solid.

^1^H-NMR (400 MHz, CDCL_3_) *δ*: 4.08 (2H, s), 7.27–7.48 (3H, m), 7.64(1H, s).

ESI-MS: *m*/*z* = 178 (M+H)^+^.

#### 4.3.3 Synthesis of 2-(3-(aminomethyl)phenoxy)acetic acid

To a solution of 2-(3-cyanophenoxy)acetic acid (100 mg, 0.564 mmol) in methanol (1.2 ml), acetic acid (1.2 ml) and Pd-C (10 mg) were added. The mixture was stirred at room temperature under H_2_ gas for 4 h. The reaction mixture was filtered and the filtrate was evaporated *in vacuo* to give the desired product (138 mg, 99%) as white solid.

^1^H-NMR (400 MHz, CDCL_3_) *δ*: 4.36 (2H, s), 4.66 (2H, s), 6.79–7.01 (3H, m), 7.15 (1H, s).

ESI-MS: *m*/*z* = 182 (M+H)^+^.

#### 4.3.4 Synthesis of 2,2,2-trichloroethyl (5-(furan-2-yl)-1,3,4-oxadiazol-2-yl)carbamate

To a solution of 5-(furan-2-yl)-1,3,4-oxadiazol-2-amine (3.02 g, 20 mmol) in THF (80 ml), 1,4-dioxane (40 ml), N-ethyl-N-isopropylpropan-2-amine (3.88 g, 30 mmol), and 2,2,2-trichloroethyl carbonochloridate (4.45 g, 21 mmol) were added. The mixture was stirred at room temperature for 20 h. The reaction mixture was evaporated under reduced pressure. The residue was purified by flash chromatography on silica gel to afford the product (5.11 g, 78%) as white solid.

^1^H-NMR (400 MHz, CDCL_3_) *δ*: 4.84 (2H, s), 6.68 (1H, dd, *J* = 1.5, 7.5 Hz), 7.08 (1H, dd, *J* = 1.5, 7.5 Hz), 7.86 (1H, dd, *J* = 1.5, 7.5 Hz), 9.15 (1H, bs).

ESI-MS: *m*/*z* = 325, 327 (M + H)^+^.

#### 4.3.5 Synthesis of 2-(3-((3-(5-(furan-2-yl)-1,3,4-oxadiazol-2-yl)ureido)methyl)phenoxy)acetic acid (Ligand1)

To a solution of 2,2,2-trichloroethyl (5-(furan-2-yl)-1,3,4-oxadiazol-2-yl)carbamate (0.098 g, 0.3 mmol) and 2-(3-(aminomethyl)phenoxy)acetic acid (0.072 g, 0.3 mmol) in acetonitrile (1.5 ml), N-ethyl-N-isopropylpropan-2-amine (0.081 g, 0.63 mmol) was added. The mixture was stirred at 333 K for 12 h. The reaction mixture was cooled to room temperature and evaporated under reduced pressure. The residue was purified by flash chromatography on silica gel to afford the product (0.090 g, 84%) as white solid.

^1^H-NMR (400 MHz, DMSO-d_6_) *δ*: 4.38 (2H, d, J = 5.6 Hz), 4.65 (2H, s), 6.78 (2H, tt, *J* = 5.12, 2.56 Hz), 6.91 (2H, tt, *J* = 5.6, 8.0 Hz), 7.18 (1H, td, *J* = 2.2, 1.22 Hz), 7.25 (1H, t, *J* = 7.93 Hz), 8.01 (2H, m, *J* = 1.6 Hz), 11.6 (1H, brs), 13.0 (1H, brs).

ESI-MS: *m*/*z* = 359 (M+H)^+^.

### 4.4 Expression of Keap1

We have expressed the Kelch domain of human Keap1 (residues 321–609) in *Escherichia coli* system using a modified protocol from the original method described by Li et al. [50]. A hexahistidine tag comprising 20 residues was added to the N-terminus. The plasmid encoding Keap1 was digested with *Nde*I and *Bam*HI, and the fragment was inserted into the expression vector pET15b (Novagen) linearized with *Nde*I and *Bam*HI. Luria–Bertani medium containing carbenicillin was inoculated with a single colony of *E. coli* BL21(DE3) (Novagen) carrying recombinant plasmid and grown at 310 K with vigorous shaking. Plusgrow medium (Nacalai tesque) containing carbenicillin was inoculated with resulting Luria–Bertani medium and grown at 310 K to reach an optical density of 0.8 at 600 nm. Then, the Keap1 expression was induced by the addition of IPTG to a final concentration of 0.4 mM at 293 K. The cells were harvested by centrifugation at 5000*g* for 10 min and stored at 193 K.

### 4.5 Purification

The cell pellets were treated with BugBuster HT Protein Extraction Reagent (Novagen). The soluble fraction was applied onto a His-Trap HP column (GE healthcare) equilibrated with the binding buffer [500 mM sodium chloride, 20 mM sodium phosphate buffer pH 7.4]. The column was washed with 2% Buffer A [1 M imidazole, 500 mM sodium chloride, 20 mM sodium phosphate buffer pH 7.4], and the His-tagged protein was eluted with 30% Buffer A. The eluate was concentrated by ultrafiltration using an Amicon Ultra-15 (Millipore, 10,000 cut-off) and applied onto a HiLoad 16/60 Superdex 200 column (GE healthcare Amersham) equilibrated with Buffer B [150 mM sodium chloride, 20 mM DTT (dithiothreitol), 20 mM Tris–HCl pH 8.3]. Peak fractions containing the protein were pooled, diluted with Buffer C [20 mM DTT, 25 mM Tris–HCl pH 8.0], and applied onto a HiTrap Q FF column (GE healthcare) equilibrated with 10% Buffer D [1 M sodium chloride, 20 mM DTT, 25 mM Tris–HCl pH 8.0]. The protein was eluted with linear gradient of 10–40% Buffer D. The purity of the protein sample was evaluated by SDS–PAGE, which showed a single band on the coomassie-stained gel. The purified protein solution was desalted and concentrated using Amicon Ultra-15 (Millipore, 10,000 cut-off) to its final composition [12 mg ml^−1^ Keap1, 150 mM sodium chloride, 20 mM DTT, 20 mM Tris–HCl pH 8.3] and stored at 193 K.

### 4.6 Crystallization, soaking and cryoprotection

Apo-form crystals were obtained using the hanging-drop vapor-diffusion method at 277 K. The crystallization drop was prepared by mixing equal volumes (1.0 μl) of the protein solution [12 mg ml^−1^ Keap1, 150 mM sodium chloride, 20 mM DTT, 20 mM Tris–HCl pH 8.3] and the precipitant solution [10% PEG6000, 10 mM magnesium chloride, 200 mM HEPES−NaOH pH 7.0]. Hexagonal rounded crystals grew in 3–7 days to an approximate size of 60 × 60 × 60 μm. To prepare the soaking form of the Keap1–Ligand1 complex, the apo-form crystals were soaked in a precipitant solution saturated with Ligand1 at 277 K for 30 min. The cocrystallization with Ligand1 was performed in the same way as the apo-form crystallization except for using different protein solution as a suspension with solid Ligand1 [8 mg ml^−1^ Keap1, 150 mM sodium chloride, 20 mM DTT, 20 mM Tris–HCl pH 8.3, 3.5 mM Ligand1] and different precipitant solution [10% PEG3350, 0.1 M calcium acetate pH7.5]. Although the actual concentration of Ligand1 in solution state in the crystallization drop was assumed to be less than 1 mM, the saturation level would be kept during crystallization because of using the suspension state. Orthorhombic crystals with low diffraction quality grew in seven days to an approximate size of 10 × 60 × 10 μm. To obtain high-resolution crystals of the cocrystallization form, these crystals were used for the seeding in which the seed crystals were added to another crystallization drop at three days after the crystallization setup. The high-resolution orthorhombic crystals grew in seven days after the seeding to an approximate size of 10 × 60 × 10 μm. All crystals for the X-ray data collection were frozen using a cryoprotectant solution consisting of 30% (*v*/*v*) ethylene glycol in the crystallization precipitant solution.

### 4.7 X-ray data collection and structure determination

X-ray diffraction data were collected at the beamline BL26B2 of SPring-8, Japan. The X-ray wavelength used was 1.0 Å with the crystal-to-detector distance of 200 mm and the oscillation angle of 1°. For both the soaking and the cocrystallization forms, complete diffraction data sets were obtained to 2.1 Å resolution at 100 K. The data were processed and scaled using the *HKL2000* program package [51]. The crystal structures were solved by the molecular replacement method using the program *MOLREP* [52] from the *CCP4* program suite [53], using an available structure of the human Keap1 Kelch domain (PDB entry 1u6d) [30] as a search model. Refinement was carried out using the programs *REFMAC*5 [54] and *CNX* (Accelrys Inc.) [55]. The structure was visualized and modified using the program *Coot* [56]. Several rounds of manual fitting and refinement were carried out through careful inspection of 2*F*_o_ − *F*_c_ and *F*_o_ − *F*_c_ electron-density maps. The stereochemical quality of the final structures was checked using the program *PROCHECK* [57]. The statistics from crystallographic analysis are given in Table 1. The superposition of models was performed using the program *LSQKAB* [58] from the *CCP4* suite. The visualization of molecules in figures was prepared using the program *Quanta 2000* (Accelrys Inc.) for Figs. 3–5, and the program *Discovery Studio* (Accelrys Inc.) for Fig. 7.

### 4.8 Molecular dynamics calculations

The molecular dynamics (MD) simulation was performed using the program *Discovery Studio* v4.0.100.13344 (Accelrys Inc.) using the CHARMm force field [59]. The atomic coordinates of 3vng and 3vnh are read into *Discovery Studio* without crystal water molecules. The protonation state was estimated using the p*K* prediction function of *Discovery Studio*; the total charge of molecules including Ligand1 was set to −5 at pH 7.4. The system in a truncated octahedron cell adopted the periodical boundary condition with the molecule-boundary distance of 20 Å. The system was then explicitly solvated and neutralized by adding Na^+^ and Cl^−^ ions to reach an ionic strength of 0.145. Each cell contained: 4334 protein atoms, 39 ligand atoms, 46 Na^+^ ions, 41 Cl^−^ ions and 46,560 water atoms for the soaking form (3vnh); 4334 protein atoms, 39 ligand atoms, 47 Na^+^ ions, 42 Cl^−^ ions and 47,430 water atoms for the cocrystallization form (3vng). The simulation protocol was composed of six sequential stages: the steepest descent minimization with the target gradient of 1.0 kcal mol^−1^ Å^−1^; the first heating stage from 0 to 10 K for 0.1 ps with the step duration of 0.1 fs; the first equilibration stage at 10 K for 1 ps with the step duration of 0.1 fs; the second heating stage from 50 to 300 K for 4 ps with the step duration of 2 fs; the second equilibration stage at 300 K for 10 ps with the step duration of 2 fs; the final NPT production stage using the leap-frog integrator at 300 K for 20 ns with the reference pressure of 1.0 atm and with the step duration of 2 fs. The SHAKE condition [60] was off in the first three stages whereas it was applied in the later stages. The particle-mesh Ewald method [61] was selected for the electrostatic calculation throughout the stages. The coordinates of the trajectory were recorded every 20 ps. The numbers of processors used for the calculation were 8 or 16. Unless otherwise noted, the parameter was set to the default value in *Discovery Studio*. The temperature fluctuation during the production stage was comparable to that estimated from the degree of freedom of the system. The number of interatomic contacts in the MD structure was calculated using the program *CONTACT* in the CCP4 suite [53]. The cluster analysis was performed using Perl scripts based on the method described by Daura et al. [45] where a series of non-overlapping clusters was obtained. First, Rmsd from a superposition of relevant atoms was calculated between all pairs of structures. Then, for each structure, the number of the other neighbor structures with the Rmsd value less than a cutoff value was calculated. The cutoff value of Rmsd was determined by a searching around 70% of the average intratrajectory Rmsd.

## Author contributions

MS, HS, RS, NN, TA, RT and NK planned experiments. MS, HS, TT, YM, HN, NY, HI, YY and NK performed experiments and/or analyzed data. MS, RT and NK wrote the paper.

## Figures and Tables

**Table 1:** Statistics from crystallographic analysis.

Crystal form	Soaking	Cocrystallization
Data collection		
Space group	*P*6_5_22	*P*2_1_2_1_2_1_
Unit-cell parameters (Å)	*a *= 85.368, *c *= 145.903	*a *= 51.328, *b *= 66.514, *c *= 77.765
Resolution range (Å)	40–2.10 (2.18–2.10)	40–2.10 (2.18–2.10)
No. of unique reflections	19,075 (1859)	16,129 (1568)
Redundancy	32.1 (32.3)	6.7 (6.8)
Completeness (%)	100.0 (100.0)	100.0 (100.0)
<*I*/*σ*(*I*)>	40.3 (12.8)	31.9 (19.4)
a*R*_merge_ (%)	9.5 (37.1)	5.3 (8.4)
Wilson *B* value (Å^2^)	20.5	17.4

Refinement		
Resolution range (Å)	40–2.10 (2.23–2.10)	40–2.10 (2.23–2.10)
No. of reflections	18,954 (3061)	16,089 (2631)
b*R*_cryst_/*R*_free_ (%)	20.1 (23.6)/21.1 (24.7)	19.6 (19.6)/22.5 (23.2)
Protein <*B*>(Å^2^)/No. of atoms	20.5/2217	14.1/2217
Ligand <*B*>(Å^2^)/No. of atoms	37.6/26	15.8/26
Water <*B*>(Å^2^)/No. of atoms	35.6/247	34.7/407
Total <*B*>(Å^2^)/No. of atoms	22.2/2490	17.3/2650
Rmsd bond lengths (Å)	0.012	0.011
Rmsd bond angles (°)	1.70	1.60
Ramachandran plot (%)		
Most favoured	88.7	89.1
Additional allowed	10.5	10.5
Generously allowed	0.8	0.4
Disallowed	0.0	0.0
PDB code	3vnh	3vng

a *R*_merge_ = ∑*_hkl_* ∑*_i_*|*I_i_*(*hkl*) − <*I*(*hkl*)>|/∑*_hkl_* ∑*_i_ I_i_*(*hkl*), where *I_i_*(*hkl*) is the *i*th observation of reflection *hkl* and < *I*(*hkl*)> is the weighted average intensity for all observations *i* of reflection *hkl*.

b *R*_cryst_ = ∑*_hkl_*||*F*_obs_| − |*F*_calc_||/∑*_hkl_*|*F*_obs_|, where |*F*_obs_| and |*F*_calc_| are the observed and calculated structure-factor amplitudes, respectively. *R*_free_ was calculated with 5% of the reflections chosen at random and omitted from refinement. Values in parentheses are for the outermost shell.

**Table 2:** Recognition of Ligand1 by Keap1.

Soaking			Cocrystallization			Type	Distance (Å)
Keap1	Water	Ligand1	Keap1	Water	Ligand1		
Tyr334 C^ε2^		CAZ				NP	3.19
C^ζ^		CAY				NP	3.37
O^η^		CAY				NP	3.30
O^η^		CAI				NP	3.14
O^η^		CAD				NP	3.33
			Tyr334 C^γ^		CAL	NP	3.34
			O^η^		NAO	HB	3.07

			Arg336 N^ε^		CAD	NP	3.25
			N^ε^		CAI	NP	3.34
			N^ε^		CAF	NP	3.39
			O	W413		HB	2.66
				W413	NAP	HB	2.50

Ser363 O^γ^		NAM				NP	3.39
O^γ^		OAB				NP	3.40
			Ser363 C^β^		OAA	NP	3.10
			O^γ^		OAA	HB	2.57
			O^γ^		CAT	NP	3.21
			O^γ^		OAC	NP	3.31

Arg380 N^η2^		NAN				NP	3.07
			Arg380 N^ε^		OAC	HB	2.88
			C^ζ^		OAC	NP	3.30
			N^η2^		OAC	HB	2.88
			N^η2^		OAQ	NP	3.22

Asn382 C^γ^		OAR				NP	3.02
O^δ1^		OAR				NP	2.91
O^δ1^		CAF				NP	3.03
			Asn382 C^β^		OAC	NP	3.21
			N^δ2^		OAC	HB	3.05
			N^δ2^		OAQ	NP	3.31

Asn414 O^δ1^	W445					HB	2.67
	W445	OAB				HB	2.58
			Asn414 O^δ1^	W433		HB	2.81
				W433	OAA	HB	3.07

Arg415 N^η1^		OAA				ES	3.51
N^η1^		OAQ				NP	3.05
N^η2^		NAP				NP	3.08
N^η2^		CAX				NP	3.20
N^η2^		CAU				NP	3.26

Arg483 N^ε^		OAC				ES	3.47
N^η2^		OAC				ES	3.44

Ser508 C^β^		OAC				NP	3.14
O^γ^		OAC				HB	2.74
O^γ^		CAT				NP	3.12

Ala556 C^β^		CAV				NP	3.39
			Ser602 O^γ^	W424		HB	2.79
				W424	OAA	HB	2.90
Gly603 C^α^		CAK				NP	3.11

Interactions are classified in three types: HB as a hydrogen bond with a distance not greater than 3.4 Å (angle considered); NP as a nonpolar interaction with a distance not greater than 3.4 Å; ES as an electrostatic interaction with a distance not greater than 4.0 Å.

**Table 3:** Keap1–Ligand1 contacts in MD trajectories.

	Keap1	Ligand1	<Distance> (Å)	Number of contacts	Frequency (%)
**Soaking form**	**Number of atoms used for superposition: 53 Rmsd cutoff used for clustering: 0.9 Å**				
All	Number of structures: 500 Total number of contacts: 4857 (9.71 contacts/structure) <Rmsd>: 1.232 Å (Intratrajectory), 2.550 Å (3vnh-trajectory)				
	Arg483 N^η2^	OAA	2.83	485	97.0
	Arg415 N^η1^	OAB	2.74	476	95.2
	Arg483 N^η2^	OAC	2.89	399	79.8
	N^η2^	CAT	3.20	381	76.2
	Arg415 C^δ^	OAB	3.13	380	76.0
	N^η1^	NAM	3.12	317	63.4
	Arg483 N^η1^	OAC	2.80	306	61.2
	N^η1^	OAA	2.88	263	52.6
	Ser508 C^β^	OAA	3.14	220	44.0
	Arg483 C^ζ^	OAA	3.25	190	38.0
	C^ζ^	OAC	3.25	146	29.2
	Ser508 C^β^	OAC	3.17	141	28.2
	Arg483 N^η1^	CAT	3.23	103	20.6

Major cluster	Number of structures: 204 Total number of contacts: 1916 (9.39 contacts/structure) <Rmsd>: 0.937 Å (Intratrajectory), 2.476 Å (3vnh-trajectory), 2.350 Å (3vnh-892nd)				
	Arg415 N^η1^	OAB	2.75	203	99.5
	Arg483 N^η2^	OAA	2.81	203	99.5
	Arg415 C^δ^	OAB	3.13	178	87.3
	Arg483 N^η2^	OAC	2.91	152	74.5
	N^η2^	CAT	3.21	146	71.6
	N^η1^	OAC	2.79	130	63.7
	Arg415 N^η1^	NAM	3.12	127	62.3
	Arg483 N^η1^	OAA	2.90	107	52.5
	Ser508 C^β^	OAA	3.12	101	49.5
	Arg483 C^ζ^	OAA	3.24	89	43.6
	Ser508 C^β^	OAC	3.13	66	32.4
	Phe478 C^δ2^	OAC	3.19	56	27.5
	Arg483 C^ζ^	OAC	3.26	56	27.5
	Phe478 C^δ2^	OAA	3.20	49	24.0
	Arg483 N^η1^	CAT	3.24	43	21.1

Minor cluster	Number of structures: 70 Total number of contacts: 734 (10.49 contacts/structure) <Rmsd>: 0.933 Å (Intratrajectory), 2.496 Å (3vnh-trajectory), 2.453 Å (3vnh-774th)				
	Arg415 N^η1^	OAB	2.75	70	100.0
	Arg483 N^η2^	OAA	2.88	67	95.7
	N^η2^	OAC	2.82	65	92.9
	Arg415 N^η1^	NAM	3.08	62	88.6
	Arg483 N^η2^	CAT	3.20	62	88.6
	Arg415 C^δ^	OAB	3.14	57	81.4
	Arg483 N^η1^	OAA	2.81	39	55.7
	N^η1^	OAC	2.81	36	51.4
	C^ζ^	OAC	3.22	27	38.6
	Ser508 C^β^	OAA	3.18	27	38.6
	Arg483 C^ζ^	OAA	3.24	25	35.7
	Ser508 C^β^	OAC	3.19	18	25.7
	C	OAA	3.15	16	22.9
	C	OAC	3.19	16	22.9
	Gly509N	OAA	3.21	14	20.0
					
**Cocrystallization form**	**Number of atoms used for superposition: 68 Rmsd cutoff used for clustering: 2.8 Å**				
All	Number of structures: 500 Total number of contacts: 4222 (8.44 contacts/structure) <Rmsd>: 4.179 Å (Intratrajectory), 4.808 Å (3vng-trajectory)				
	Arg380 N^η2^	OAA	2.91	218	43.6
	N^η2^	OAC	2.91	195	39.0
	N^η2^	CAT	3.18	180	36.0
	Tyr334 O^η^	OAC	2.99	175	35.0
	O^η^	CAT	3.11	169	33.8
	Arg336 N^η1^	OAC	2.98	165	33.0
	Tyr334 O^η^	OAA	3.02	146	29.2
	Arg336 N^η1^	CAT	3.19	145	29.0
	N^η2^	OAQ	3.04	131	26.2
	N^η1^	OAA	3.00	126	25.2

Major cluster	Number of structures: 180 Total number of contacts: 1845 (10.25 contacts/structure) <Rmsd>: 2.543 Å (Intratrajectory), 3.995 Å (3vng-trajectory), 3.831 Å (3vng-658th)				
	Arg380 N^η2^	OAA	2.92	119	66.1
	Tyr334 O^η^	CAT	3.10	109	60.6
	O^η^	OAC	2.99	93	51.7
	Arg380 N^η2^	OAC	2.88	92	51.1
	Tyr334 O^η^	OAA	3.02	81	45.0
	Arg380 N^η2^	CAT	3.18	80	44.4
	Arg336 N^η1^	OAC	3.01	61	33.9
	N^η1^	CAT	3.24	57	31.7
	N^η1^	OAQ	3.14	57	31.7
	Asn414 N^δ2^	OAC	2.93	53	29.4
	Arg380 N^ε^	OAC	2.96	52	28.9
	Arg415 N^η2^	OAA	2.85	50	27.8
	Arg336 N^η1^	CAL	3.22	44	24.4
	N^η2^	CAJ	3.25	44	24.4
	Asn382 N^δ2^	CAH	3.22	39	21.7
	Arg336 N^η1^	OAA	3.02	37	20.6
	Arg380 C^ζ^	OAC	3.29	37	20.6
	Arg415 N^η2^	CAT	3.17	37	20.6
	N^η1^	OAA	2.92	36	20.0

Second cluster	Number of structures: 79 Total number of contacts: 659 (8.34 contacts/structure) <Rmsd>: 2.412 Å (Intratrajectory), 5.035 Å (3vng-trajectory), 4.793 Å (3vng-842nd)				
	Arg336 N^η2^	OAQ	2.99	54	68.4
	N^η1^	OAC	2.96	37	46.8
	N^η2^	OAC	2.85	37	46.8
	Tyr334 O^η^	OAC	2.91	28	35.4
	Arg336 N^η1^	CAT	3.13	28	35.4
	N^η1^	OAA	3.04	28	35.4
	N^η2^	CAT	3.22	26	32.9
	N^η2^	OAA	2.85	20	25.3
	N^η1^	OAQ	3.20	19	24.1
	C^ζ^	OAC	3.26	18	22.8
	O	CAG	3.18	17	21.5
	Arg380 N^η2^	OAC	2.91	17	21.5
	Gln337 N^ε2^	OAB	3.02	16	20.3

Third cluster	Number of structures: 63 Total number of contacts: 595 (9.44 contacts/structure) <Rmsd>: 2.390 Å (Intratrajectory), 5.489 Å (3vng-trajectory), 5.277 Å (3vng-597th)				
	Arg380 N^η2^	OAA	2.89	50	79.4
	N^η2^	CAT	3.19	40	63.5
	Tyr334 O^η^	CAT	3.17	32	50.8
	O^η^	OAA	2.94	32	50.8
	O^η^	OAC	3.04	26	41.3
	Arg380 N^ε^	OAC	2.96	25	39.7
	Arg415 N^η2^	OAA	2.79	22	34.9
	Arg380 N^η2^	OAC	2.99	21	33.3
	Asn414 N^δ2^	OAC	2.93	21	33.3
	Arg380 N^η2^	OAQ	3.04	20	31.7
	Tyr334 C^ε1^	OAA	3.26	19	30.2
	Arg336 N^η1^	OAC	2.96	18	28.6
	N^η2^	OAC	3.07	14	22.2
	Arg380 N^η2^	CAL	3.26	14	22.2
	Arg336 N^η2^	OAQ	3.21	13	20.6
	Arg380 C^ζ^	OAC	3.31	13	20.6

For selected structures in the latter half of 20.014 ns MD trajectory from 10.014 ns to 19.994 ns comprising 500 structures from 501st to 1000th, the protein–ligand contacts with interatomic distances not greater than 3.4 Å were counted and listed after a descending sort by the number of contacts. Only major contacts with appearance frequency values not less than 20% are shown. For the atomic superposition, a pair of protein–ligand atoms of which shortest interatomic distance in the 500 structures from 501st to 1000th was not greater than 3.4 Å was selected, except for the atoms with possibility of flipping in the MD simulation: C^δ1^, C^δ2^, C^ε1^ and C^ε2^ of tyrosine/phenylalanine; OAA and OAC of Ligand1.

## Supplementary data

Supplementary data 2

Supplementary data 3 **Fig. S1.** AlphaScreen assay to confirm the Keap1–Ligand1 interaction. **Fig. S2.** Electron density maps for Ligand1 in the complex crystals. **Fig. S3.** Statistics from the MD simulation for 20.014 ns. **Fig. S4.** Stereo representations of clusters observed in MD trajectories.
