# Supplementary material for: Multiple binding modes of a small molecule to human Keap1 revealed by X-ray crystallography and molecular dynamics simulation
Source: FEBS Open Bio. 2015 Jun 30;5:557–70. doi: 10.1016/j.fob.2015.06.011 (PMC4506958; doi:10.1016/j.fob.2015.06.011)
Supplement: Supplementary data 3 — Fig. S1. AlphaScreen assay to confirm the Keap1–Ligand1 interaction. Fig. S2. Electron density maps for Ligand1 in the complex crystals. Fig. S3. Statistics from the MD simulation for 20.014 ns. Fig. S4. Stereo representations of clusters observed in MD trajectories. [file mmc3.pdf]

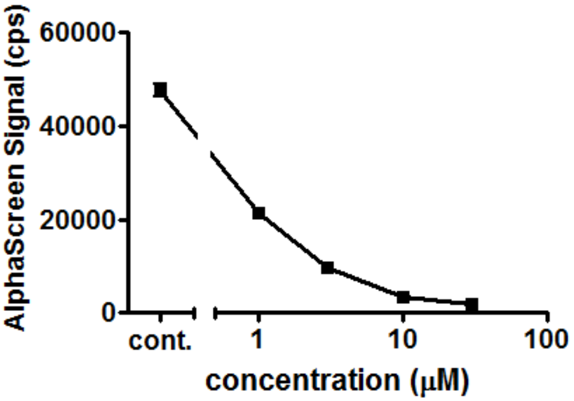

Ligand1		
μM	Exp1	Exp2
0.0	46558.0	48489.0
1.0	21179.0	21784.0
3.0	10222.0	9143.0
10.0	3094.0	3493.0
30.0	2087.0	1912.0

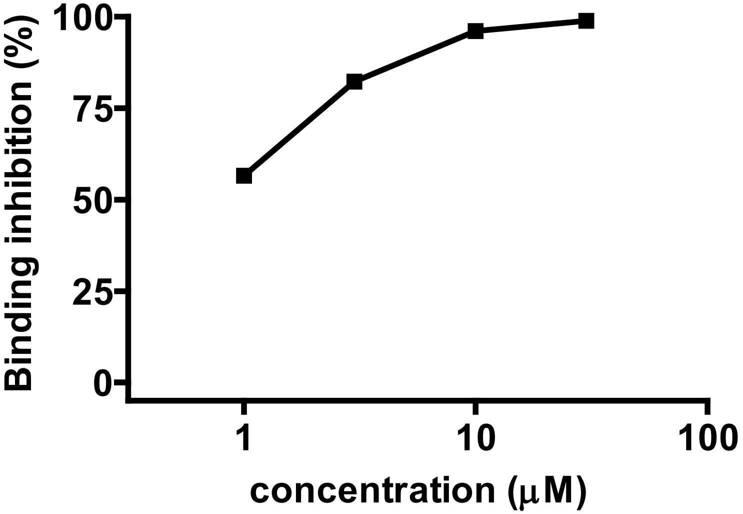

AlphaScreen assay to confirm the Keap1-Ligand1 interaction. An additional assay using AlphaScreen (PerkinElmer Inc.), a bead-based, amplified luminescent proximity homogeneous assay, was performed to evaluate the effect of Ligand1 on the interaction between the Nrf2 peptide and the Kelch domain of Keap1. The Nrf-2 ETGE peptide used was a 16-mer peptide containing amino acids 69–84 (AFFAQLQLDEETGEFL) of Nrf-2. The His-tagged Kelch domain and the biotin-tagged ETGE peptide were immobilized on the Ni-acceptor beads and the streptavidin-donor beads, respectively. The assay was performed in duplicate by adding an aqueous solution of Ligand1 at various concentrations to the beads mixture (left panel). A relative binding inhibition values were calculated assuming that the alphascreen signals from the solution without Ligand1 and from the 1 μM ETGE peptide solution corresponded to 0% and 100% of inhibitions, respectively (right panel). We detected the inhibitory effect of Ligand1 on the Keap1-Nrf2 interaction.

3vnh

3vng

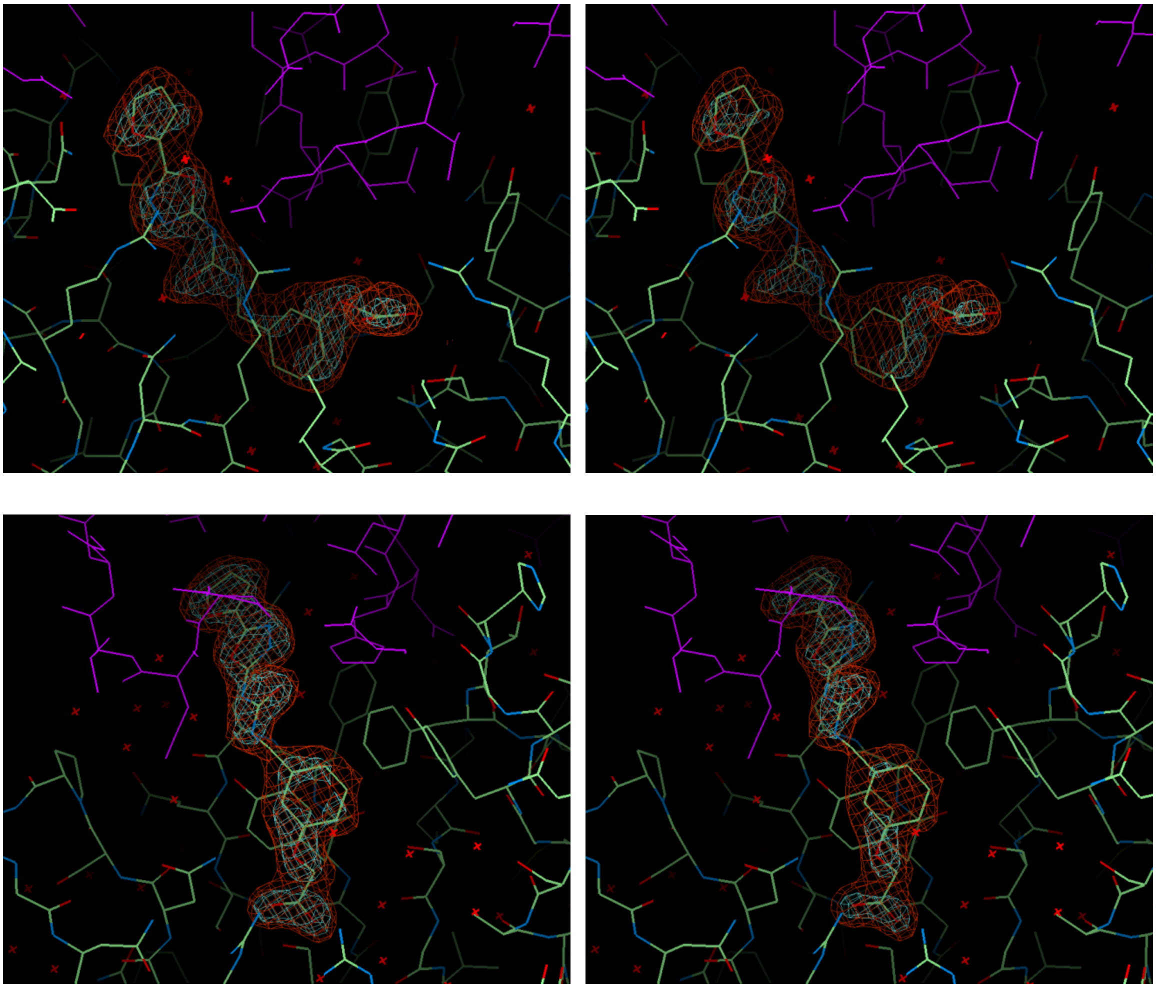

Final map

Annealed omit map

Electron density maps for Ligand1 in the complex crystals. The molecules of Keap1 and Ligand1 are depicted as stick models for the soaking form (top panels) and the cocrystallization form (bottom panels). Perspectives are similar to those used in the Fig. 5 in text. Atoms in the asymmetric unit are shown with the atom-type colouring, whereas the symmetry-related atoms are coloured magenta. Two different types of $2Fo-Fc$ electron density maps contoured at 1.0σ (orange) and 2.0σ (aqua) are overlaid around the Ligand1 models: the final map from the refined model (left panels) and the annealed omit map (right panels). The annealed omit map was calculated using the program *CNX* (Accelrys Inc.) from all atoms of final model except for those of Ligand1; a standard slow-cool protocol with the initial temperature of 2500 K was used. This figure was prepared using the program *Quanta 2000* (Accelrys Inc.).

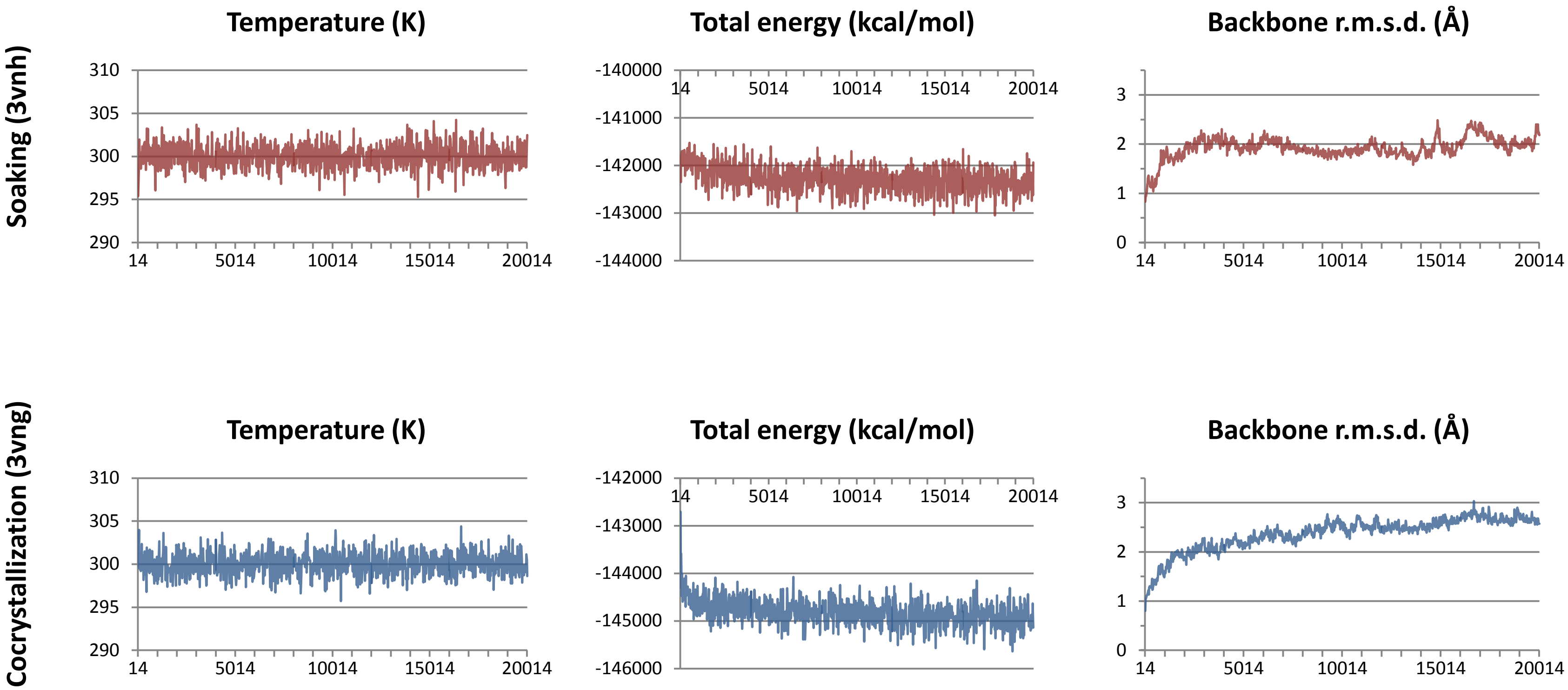

Statistics from the MD simulation for 20.014 ns. The temperature, the total energy, and the backbone rmsd from a superposition onto the crystal structure are plotted versus the simulation time in ps for the soaking form (3vnh) and for the cocrystallization form (3vng).

Supplemental Fig. S4 Satoh *et al.*

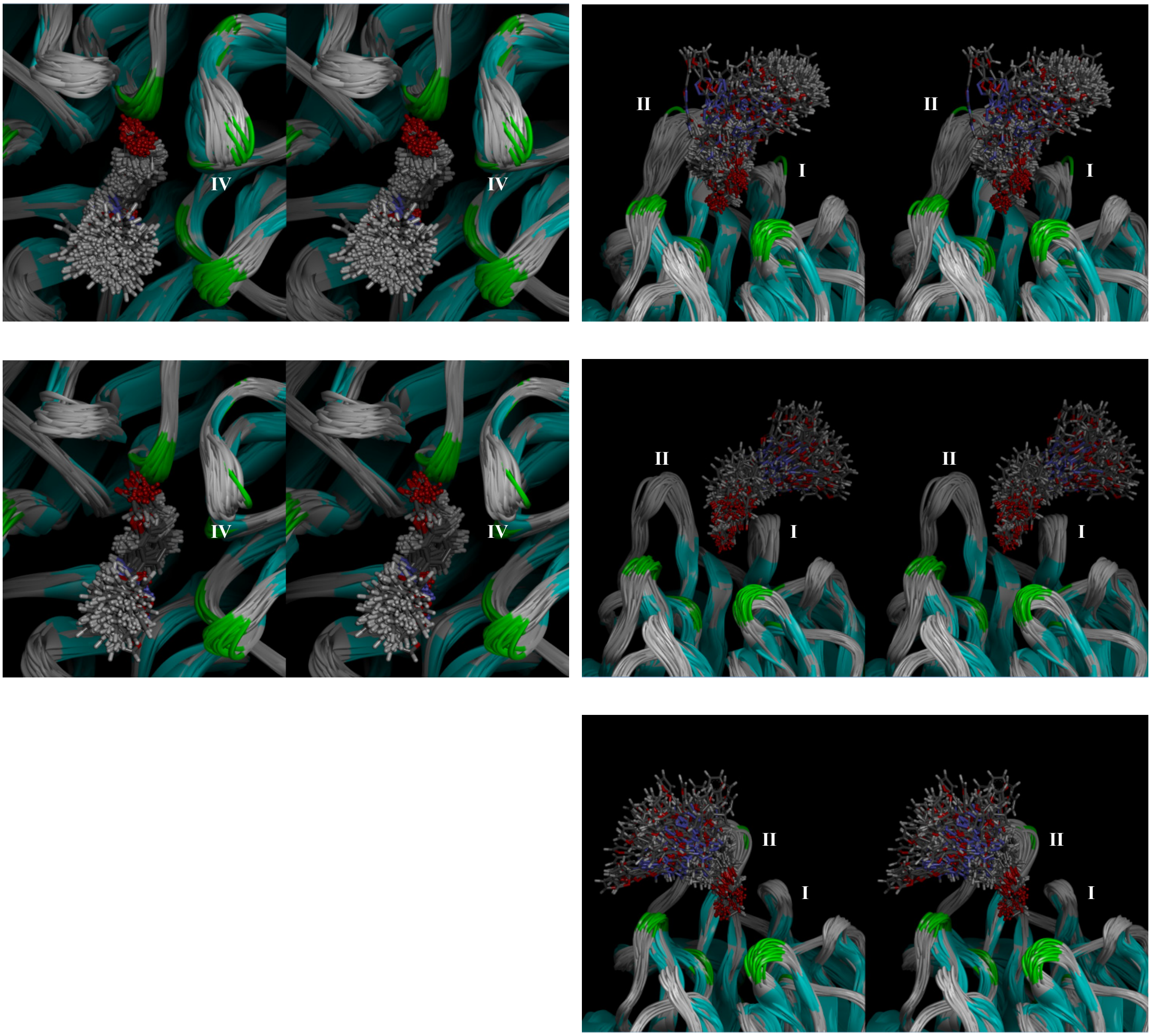

Stereo representations of clusters observed in MD trajectories. The clusters shown are: the major cluster sharing 204 structures with the 892nd as the center (top, left) and the minor cluster sharing 70 structures with the 774th as the center (bottom, left) from the soaking form; the major cluster sharing 180 structures with the 658th as the center (top, right), the second cluster sharing 79 structures with the 842nd as the center (middle, right) and the third cluster sharing 63 structures with the 597th as the center (bottom, right), from the cocrystallization form. All structures comprising a cluster are superimposed at corresponding backbone atoms. The Keap1 domain and the Ligand1 molecule are depicted as a ribbon drawing with the secondary-structure-based colouring and a stick model with the atom-type colouring, respectively. The β-hairpins in the first, the second and the fourth blades are indicated as Roman numerals. This figure was prepared using the program *Discovery Studio* (Accelrys Inc.).
